# Molecular Maneuvers and Host Sabotage: A Comprehensive Review of CSFV’s Multifaceted Strategies to Subvert Immune Defenses and Cellular Metabolism

**DOI:** 10.3390/v18030301

**Published:** 2026-02-28

**Authors:** Wenqiang Sun, Lu Xu, Jiaxin Li

**Affiliations:** 1 National Classical Swine Fever Reference Laboratory, China Institute of Veterinary Drug Control, Beijing 100018, China; sunwenqiangsyfq@163.com (W.S.); xulu777_ivdc@163.com (L.X.); 2 State Key Laboratory of Biopharmaceutical Preparation and Delivery, Institute of Process Engineering, Chinese Academy of Sciences, Beijing 100190, China

**Keywords:** CSFV, immune evasion, cellular receptors, virus–host interaction

## Abstract

Classical swine fever virus (CSFV) remains a significant threat to the global swine industry, causing a highly contagious and often fatal disease in pigs. This review comprehensively examines the molecular biology of CSFV and the intricate mechanisms it employs to establish infection. We detail the structure and functions of viral proteins, highlighting their roles in virus entry, replication, and immune evasion. A major focus is placed on the virus–host interaction, specifically how CSFV subverts host innate immune responses and hijacks critical cellular processes, including metabolism and cell death pathways. The virus strategically manipulates host cell death programs (apoptosis, mitophagy, necroptosis) and exploits intracellular transport systems to promote its propagation. Furthermore, we summarize recent advances in understanding the cellular receptors involved in CSFV entry and the role of exosomes in viral spread. This synthesis of current knowledge aims to provide a deeper insight into the pathogenesis of CSFV and identify potential vulnerabilities that could be targeted for the development of novel antiviral strategies.

## 1. Introduction

Classical swine fever (CSF), caused by classical swine fever virus (CSFV), is a highly contagious and economically significant disease of swine, classified as a notifiable disease by the World Organization for Animal Health (OIE). The disease is characterized by a wide range of clinical signs, from acute forms with high fever, hemorrhagic diathesis, and high mortality, to chronic and asymptomatic forms, posing a persistent challenge to disease control and eradication programs worldwide [1,2]. The fight against CSF has historically relied on extensive vaccination with live-attenuated vaccines, such as the C-strain, which have been instrumental in controlling major outbreaks [3]. The prevalence of acute, classical CSF has decreased in many regions using vaccination, but it has been replaced by atypical, chronic, or persistent forms of the disease [4]. This shift underscores the remarkable adaptability of CSFV and highlights a critical gap in our understanding—namely, how the virus successfully evades both immune-mediated clearance and vaccination-induced protection at a fundamental level.

Addressing this gap necessitates a deep dive into the molecular interface of the virus–host interaction. The molecular pathogenesis of CSFV is complex and multifaceted, revolving around its sophisticated ability to subvert, hijack, and reprogram host cellular processes. A comprehensive understanding of these mechanisms is not only of fundamental virological interest but is also crucial for developing next-generation antiviral strategies. Therefore, this review aims to synthesize current knowledge on the molecular biology of CSFV, with a particular emphasis on the mechanisms that underpin its successful infection cycle and pathogenesis. We will first outline the viral structure and the functions of its encoded proteins. This will be followed by a detailed analysis of the multi-receptor viral entry process. Subsequently, we will systematically review the strategies employed by CSFV to suppress host innate and adaptive immune responses, to exploit cellular resources and metabolism, and to precisely manipulate key cell death pathways, including apoptosis, necroptosis, and autophagy. Finally, by integrating these aspects, this review seeks to provide a holistic perspective on the molecular determinants of CSFV persistence and pathogenicity, thereby informing future research directions for improved disease control.

## 2. Virus Structure of CSFV

**Figure 1:**
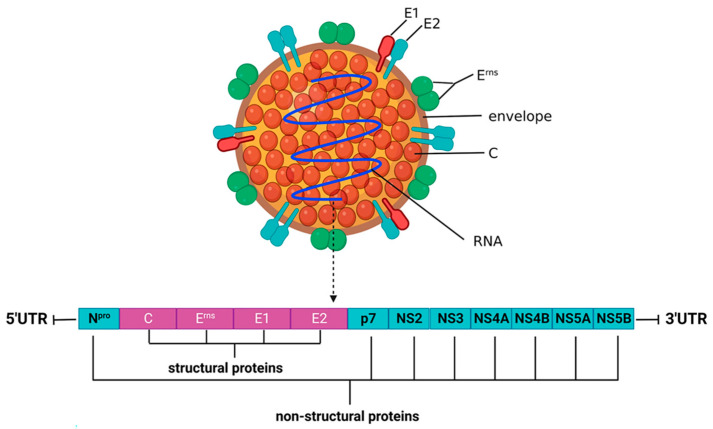
Schematic of CSFV particle and organization of CSFV genome [8].

CSFV is an enveloped, single-stranded, positive-sense RNA virus belonging to the *Pestivirus* genus within the *Flaviviridae* family [5]. The virion is spherical and measures approximately 40–60 nm in diameter, containing the icosahedral symmetric nucleocapsid. It has a genome length of about 12.3 kb, consisting of a large open reading frame (ORF), a 5′-untranslated region (5′-UTR) and a 3′-untranslated region (3′-UTR), and is encapsulated in the nucleocapsid (Figure 1). CSFV ORF encodes a single precursor polyprotein (about 437.8 kDa) comprising 3898 amino acids (aa). The large polyprotein is cleaved by cellular and viral proteases into 12 proteins, including four structural proteins [core protein (C) and envelope glycoproteins (E^rns^, E1, and E2)] and eight non-structural proteins (Npro, p7, NS2, NS3, NS4A, NS4B, NS5A, and NS5B). The sequence of viral proteins from N-terminus to C-terminus was found to be Npro-C-E^rns^-E1-E2-p7-NS2-NS3-NS4A-NS4B-NS5A-NS5B [6,7]. These viral proteins stick to their respective roles and coordinate with each other and/or interact with host cell proteins to enable the virus to successfully become an invading pathogen.

## 3. Functions of Viral Encoded Proteins

### 3.1. Structural Protein

The structure proteins comprise C, E^rns^, E1 and E2. The envelope-associated glycoproteins E^rns^, E1 and E2 are anchored within the viral lipid envelope, while capsid protein C assembles to form an icosahedral nucleocapsid. This structure determines their functions: E^rns^, E1 and E2 are responsible for CSFV invasion of host cells, and C protein provides protection for viral nucleic acids. Of course, these features are just the tip of the iceberg because they are all multifunctional proteins.

The C protein is a small and relatively conserved protein rich in basic amino acids (lysine and arginine) [9]. The mature C protein, comprising 86 amino acids with a molecular weight of 14 KDa, is located between N^pro^ and E^rns^. Its N terminus is cleaved by autocatalysis of the N^pro^ protein, while the C terminus is cleaved by cell signal peptidase (SP) [10,11,12]. It is mainly involved in nucleocapsid formation and virion assembly [13,14,15]. It was found that C protein enhanced RNA synthesis via the CSFV polymerase NS5B protein [16]. Interaction of C protein with hemoglobin subunit β (HB) was found to inhibit the HB-mediated RIG-I interferon (IFN) signaling pathway and promote the replication of CSFV [17]. Thus, C protein seems to be an anti-host innate immune protein.

The E^rns^ protein is essential for CSFV replication but dispensable for infection [18]. It plays a crucial role in viral adsorption by mediating the initial attachment of the virus to host cells through interactions with heparan sulfate and the laminin [19,20,21]. Furthermore, as an important immunogenic glycoprotein, E^rns^ stimulates the production of neutralizing antibodies. It exists in two forms: a membrane-anchored form, which is attached via a C-terminal amphipathic helix, and a soluble form exhibiting ribonuclease activity that contributes to viral immunosuppression [22,23,24]. With a molecular weight of about 45 kDa, E^rns^ usually forms a homodimer of about 100 kDa via disulfide bonds between the carboxyl-terminal cysteine 171 of each monomer [25,26].

The E1 protein, with a molecular weight of 33 kDa, often forms disulfide-bonded E1-E2 heterodimers on the viral envelope, which are crucial for mediating viral endocytosis [27]. E1 is embedded in the viral envelope, so it cannot stimulate the production of neutralizing antibodies [28].

The E2 protein is a glycoprotein of approximately 55 kDa consisting of 373 amino acids. It is anchored to the viral envelope through a hydrophobic sequence at its C-terminus. Most of them form heterodimers of about 75 kDa with E1 via disulfide bonds, and a few form homodimers of about 100 kDa with themselves [29]. Importantly, E2 is the most immunogenic of the glycoproteins that is responsible for stimulating the production of neutralizing antibodies. Detection of antibodies against CSFV E2 is the most effective way to evaluate herd immunity. Therefore, E2 has always been the preferred target protein for the development of various new CSFV vaccines and detection technologies [30,31,32].

### 3.2. Non-Structural Protein

Non-structural proteins consist of Npro, p7, NS2, NS3, NS4A, NS4B, NS5A, and NS5B. Among the non-structural proteins, NS3, NS4A, NS4B, NS5A and NS5B are essential for viral replication, while Npro, p7 and NS2 are non-essential but play significant roles in immune evasion and viral morphogenesis.

The first protein, Npro, encoded by the viral open reading frame (ORF), is a cysteine protease with proteolytic activity that cleaves itself from the polyprotein chain and becomes a mature protein. Although not essential for viral replication [33], Npro plays an important role in anti-host cell innate immunity. It inhibits the expression of I-IFN by interacting with interferon regulatory factor 3 (IRF3) and inducing its proteasomal degradation [34,35,36].

CSFV p7 protein is a small hydrophobic polypeptide of 6–7 kDa [37]. The cleavage between E2 and p7, mediated by host signal peptidase at leucine residue 1063 of the polyprotein, is often incomplete. This results in the presence of both E2 and E2-p7 precursor forms in infected cells, although only mature E2 is incorporated into virions [38,39]. Functioning as a viroporin, p7’s pore-forming activity resides in its C-terminal transmembrane helix, facilitating viral virulence and budding [40,41,42].

NS2 is an autoprotease that mediates the cleavage between NS2 and NS3. This cleavage yields the fully processed NS3 (80 kDa) and a precursor NS2-NS3 (120 kDa) [43,44]. Once cleaved, NS2 has no role in viral replication and packaging. NS3 is a multifunctional protein with serine protease, helicase and nucleoside triphosphatase (NTPase) activities [45,46,47]. The cleavage of NS2-NS3 is a tightly regulated and critical step that enables the spatiotemporal separation of distinct viral functions. The released mature NS3 protein, in complex with NS4A, is indispensable for viral RNA replication [48]. Conversely, the uncleaved NS2-NS3 precursor, also in association with NS4A, performs a unique and essential function in the packaging and formation of infectious virions—a role that cannot be fulfilled by the processed NS3 protein alone [43]. Studies indicate that the NS2-NS3/NS4A complex is essential for the production of infectious virus particles during the late stages of infection, confirming the specific role of the uncleaved precursor in virion morphogenesis [49,50]. It is proposed that this complex may act as a scaffold to coordinate the encapsidation of the viral genome, ensuring the proper assembly of nucleocapsids prior to envelopment. This functional segregation ensures the temporal and spatial coordination of genome replication and particle assembly, which is vital for efficient viral propagation [51].

NS4A is an essential cofactor of the NS3 protease, assisting NS3 in cleavage between NS4B/NS5A and NS5A/NS5B sites. The viral RNA replication complex formed by NS4A, NS3 and NS5B participates in the synthesis of the viral genome [49]. *Flavivirus* NS4A is primarily located in the endoplasmic reticulum (ER) [52,53]. Recent studies have shown that NS4A is localized in mitochondria and achieves efficient viral replication by promoting the production of UMP [54]. NS4B is a hydrophobic protein, which colocalizes mainly with the Golgi compartment [55]. A study showed that NS4B inhibits poly (I:C) stimulation-mediated activation of the TLR3 signaling pathway in porcine monocyte-derived macrophages (pMDMs), thereby suppressing the secretion of IL-6 and IFN-β [56].

NS5A and NS5B are enzyme molecules essential for viral replication. NS5A (58 kDa) is a phosphokinase that can compete with NS3 to bind to the same sequence on the 5′UTR IRES of viral RNA, reducing the translation efficiency of IRES-initiated protein [57]. NS5A can also bind to 3′UTR of a viral genome to regulate the synthesis of viral RNA [58]. NS5B (77 kDa) has RNA-dependent RNA polymerase (RdRp) activity and is able to bind its cognate 3′UTR and initiates genome replication [59]. The N-terminal amino acid residues 63–99 and the C-terminal amino acid residues 611–642 of the NS5B can bind to NS3 and enhance the activity of RdRp [60]. In addition, NS5B can counteract the inhibitory effect of NS5A on viral transcription, ensuring efficient viral gene expression [61]. A summary of the properties and functions of both structural and non-structural CSFV proteins is provided in Table 1.

## 4. Cellular Receptors and Attachment Factors

The entry of CSFV into host cells is a multistep process orchestrated by the interactions between its three envelope glycoproteins (E^rns^, E1, and E2) and specific cellular membrane molecules. Among these, the E2 glycoprotein is established as the primary determinant of viral cell tropism, directly engaging with key receptors to mediate internalization. To date, a diverse array of host factors, including heparan sulfate (HS), laminin receptor (LamR), complement regulatory protein CD46, integrin β3, annexin II, MER tyrosine kinase (MERTK), a disintegrin and metalloproteinase 17 (ADAM17), vinculin, and the low-density lipoprotein receptor (LDLR), have been implicated in the attachment and entry of CSFV. The roles of the above involved factors are listed in Table 2.

Heparan sulfate, a negatively charged glycosaminoglycan, acts as an attachment factor for many viruses [68]. Passage of CSFV in cultured swine kidney cells was shown to cause the amino acid residue 476 of E^rns^ protein to mutate from Ser to Arg. This change enabled heparan sulfate (HS) to become the receptor of CSFV [19]. The laminin receptor (LamR) has been found to colocalize with CSFV virions on the cell membrane and interacts with CSFV E^rns^. Inhibition of LamR by anti-LamR antibodies, soluble laminin, or LamR protein significantly inhibited CSFV infection [21]. CD46 is a type I transmembrane protein that has been identified as a receptor for BVDV [69,70]. Studies have shown that CD46 is involved in CSFV infection. Blocking CD46 protein almost completely inhibited CSFV infection. However, when the virus was serially passaged in culture, blockade of CD46 had a weaker effect on CSFV infection [62]. This may be due to the mutation of CSFV after adaptation to cell culture using HS as a receptor.

Beyond these, several other host molecules facilitate the entry process. Integrin β3, a member of the integrin superfamily [71], is up-regulated after CSFV infection. Functional blockade of integrin β3 reduces viral load in infected cells, suggesting its role in mediating internalization [63]. The calcium-dependent phospholipid-binding protein Annexin II supports various cellular processes, including endocytosis [72,73]. During CSFV infection, it is also upregulated and colocalizes with E2. Neutralization of annexin II impairs viral replication, indicating its involvement in entry [64]. MERTK is a member of the TAM (TYRO3, AXL, and MERTK) receptor protein tyrosine kinases, and promotes phagocytosis and modulates innate immunity [74,75]. Research showed that MERTK can interact with E2 to facilitate entry, as evidenced by co-immunoprecipitation and SPR analysis. The inhibitory effect of the soluble MERTK ectodomain on infection further confirms its role as a functional entry receptor [65]. Similarly, a disintegrin and metalloproteinase-17 (ADAM17), also named tumor necrosis factor-α-converting enzyme (TACE), is essential for CSFV entry through its interaction with E2 [66]. Knockdown of the expression of vinculin (a component of cell–matrix adhesion and cell–cell adhesion) was found to have a significant impact on CSFV infection, suggesting that vinculin may also be one of the receptors for CSFV invasion into cells [21]. Finally, LDLR appears to influence CSFV replication, as its deficiency or antibody-mediated blockade inhibits infection. However, recombinant soluble LDLR fails to neutralize certain CSFV strains, suggesting that LDLR may function indirectly, possibly by modulating lipid metabolism crucial for the viral lifecycle, rather than acting as a classic binding receptor [67].

## 5. Suppression of Host Immune Responses

Histone acetylation is a key epigenetic mechanism that dynamically regulates immune gene expression [76]. Generally, histone acetylation relaxes chromatin structure, facilitating the access of transcription factors and RNA polymerase II to promoter regions, thereby promoting the transcription of genes involved in innate immune responses, such as type I interferons (IFNs) and pro-inflammatory cytokines [77,78]. However, CSFV subverts this pro-host mechanism to evade antiviral defenses. A well-characterized example is mediated by the viral Npro protein, a key contributor to innate immune suppression [79,80,81]. It targets the host’s epigenetic machinery by downregulating histone deacetylase 1 (HDAC1), an enzyme that typically removes acetyl groups to repress gene expression. Specifically, Npro interacts with the transcription factor specificity protein 1 (Sp1), a key regulator of HDAC1 transcription, and promotes its ubiquitin–proteasome-dependent degradation. The consequent reduction in Sp1 and HDAC1 levels disrupts normal histone deacetylation, altering the host cell’s epigenetic landscape and contributing to the suppression of antiviral gene expression, thereby facilitating viral immune evasion [82]. Additionally, Npro upregulates the acetylation of high mobility group box 1 (HMGB1), a non-histone protein, triggering its translocation from the nucleus to the cytoplasm for lysosomal degradation and consequently subverting HMGB1-mediated antiviral immunity [83]. Furthermore, CSFV infection induces the expression of indoleamine 2,3-dioxygenase 1 (IDO1) in PK-15 cells. IDO1 suppresses the NF-κB signaling pathway by promoting tryptophan metabolism, thereby creating a favorable environment for CSFV replication [84].

Beyond Npro, other non-structural proteins of CSFV also play significant roles in modulating host immunity. For instance, NS4A interacts with dihydroorotate dehydrogenase (DHODH), a rate-limiting enzyme in the de novo pyrimidine synthesis pathway. This interaction enhances DHODH activity and promotes the production of UMP, which supports efficient viral RNA synthesis. The importance of this pathway is highlighted by the fact that specific inhibition of DHODH suppresses CSFV replication, both by enhancing innate immune responses and by blocking pyrimidine synthesis [54].

The NS5A protein contributes to immune suppression by downregulating key inflammatory cytokines such as IL-1β, IL-6, and TNF-α in porcine alveolar macrophages through inhibition of the NF-κB signaling pathway [85]. Further evidence indicates that NS5A directly binds to the NF-κB essential modulator (NEMO), a regulatory subunit of the IκB kinase (IKK) complex, and mediates its proteasomal degradation, thereby further suppressing NF-κB activation [86].

**Figure 2:**
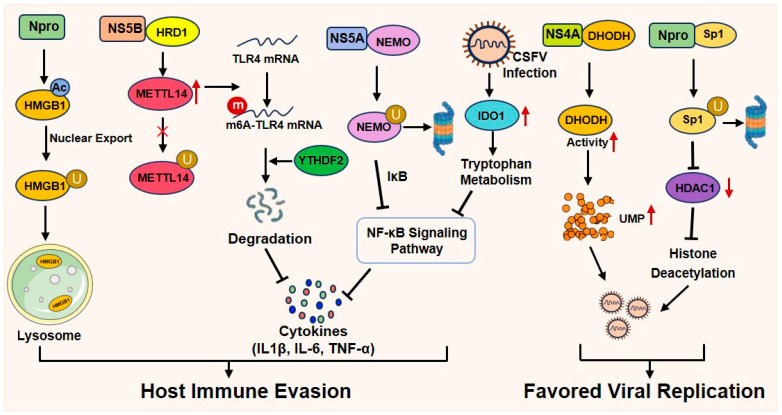
CSFV non-structural proteins: a multi-faceted strategy for host immune evasion.

CSFV also manipulates host epitranscriptomic mechanisms to evade immune surveillance. The non-structural protein 5B (NS5B) interacts with the E3 ubiquitin ligase HRD1, which normally targets the methyltransferase METTL14 for degradation. By shielding METTL14 from HRD1-mediated ubiquitination, NS5B stabilizes METTL14 and increases its intracellular expression. The elevated METTL14 levels promote m6A modification of Toll-like receptor 4 (TLR4) mRNA, recruiting the reader protein YTHDF2 and leading to TLR4 mRNA degradation. This process inhibits the TLR4-regulated NF-κB pathway and facilitates viral immune escape [87] (Figure 2).

## 6. Exploitation of Host Cell Resources

**Figure 3:**
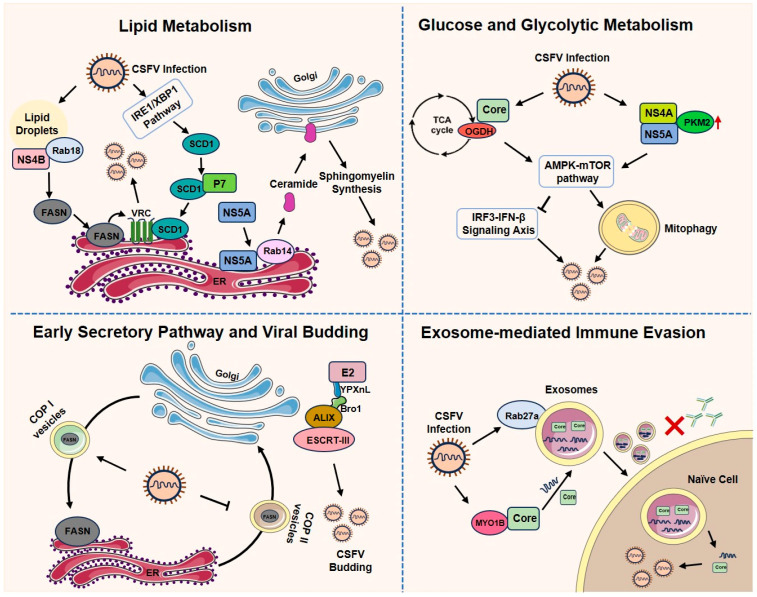
Modulation of host metabolic and secretory pathways by CSFV supports viral replication and immune evasion.

CSFV disrupts the normal metabolic processes of host cells to hijack cellular resources, thereby facilitating the synthesis of viral RNA and proteins and completing its own life cycle. This reprogramming affects multiple metabolic pathways, including lipid, glucose, and glycolytic metabolism, and exploits the host’s transport systems to promote viral propagation (Figure 3).

### 6.1. Lipid Metabolism

CSFV extensively manipulates host lipid metabolism to support its entry, replication, and assembly. The virus relies on key endocytic regulators such as Rab5, Rab7, and Rab11 GTPases for cellular invasion [88,89]. After entry, CSFV further reprograms lipid biosynthesis: the non-structural protein NS4B interacts with Rab18 on lipid droplets, upregulating fatty acid synthase (FASN) and recruiting it to the endoplasmic reticulum (ER) to promote the formation of the viral replication complex (VRC) [90]. In addition, NS5A recruits Rab14 to the ER, facilitating the transport of ceramide to the Golgi apparatus for sphingomyelin synthesis, which enhances viral particle assembly [91]. Recent studies indicate that stearoyl-CoA desaturase 1 (SCD1), a key enzyme in monounsaturated fatty acid production, is upregulated during CSFV infection through the IRE1α/XBP1 endoplasmic reticulum stress pathway. SCD1 interacts with the viral p7 protein and is recruited to the VRC, where it modulates lipid metabolism to support efficient viral replication [92].

### 6.2. Glucose and Glycolytic Metabolism

CSFV targets central carbon metabolism to ensure energy and biosynthetic precursor supply. The viral Core (C) protein interacts with α-ketoglutarate dehydrogenase (OGDH), the first rate-limiting enzyme of the tricarboxylic acid (TCA) cycle, and promotes its degradation via the AMPK-mTOR-autophagy pathway. This regulation suppresses the OGDH-dependent IRF3-IFN-β innate immune signaling axis, thus facilitating viral replication [93]. Meanwhile, CSFV infection upregulates the expression of pyruvate kinase M2 (PKM2), a critical regulatory enzyme in glycolysis, both in vitro and in vivo. PKM2 interacts with NS4A and NS5A and induces mitophagy by activating the AMPK-mTOR pathway, thereby enhancing viral proliferation [94].

### 6.3. Early Secretory Pathway and Viral Budding

CSFV co-opts the host secretory machinery to promote its replication and dissemination. The early secretory pathway, which comprises the ER and Golgi apparatus along with COP I and II vesicles, is hijacked to transport viral components. A recent study demonstrated that CSFV uses COP I vesicles to relocate FASN—a critical host factor for viral RNA synthesis—from the Golgi to the ER, while simultaneously inhibiting FASN efflux via COP II vesicles, thereby enhancing viral RNA replication [95]. During the budding stage, the virus recruits the Bro1 domain of ALIX (an ESCRT-associated protein) to recognize the YPXnL late domain motif on the CSFV E2 glycoprotein. ALIX, together with ESCRT-III, then orchestrates viral budding near the Golgi apparatus [96].

### 6.4. Exosome-Mediated Immune Evasion

Exosomes, which are small membrane-enclosed vesicles, are actively released into the extracellular space by a variety of cells. Following CSFV infection, these exosomes carry viral genomic RNA and partial structural proteins, and are capable of being internalized by recipient cells. Crucially, such exosomes can bypass CSFV-specific neutralizing antibodies, thereby establishing productive infection in naïve cells [97]. A key mechanism underlying this process involves the molecular interaction between the viral core (C) protein and the host myosin 1B (MYO1B) protein. This interaction promotes the active loading of viral components into exosomes, facilitating a non-lytic viral spread that is invisible to antibody-based immunity [98]. Moreover, CSFV infection upregulates the small GTPase Rab27a, a central regulator of multivesicular body (MVB) docking and exosome release. Inhibition of Rab27a or the exosomal pathway significantly reduces viral titers, confirming the functional importance of exosome-mediated viral egress in the CSFV life cycle [99].

## 7. Controlling the Cell Death Program

**Figure 4:**
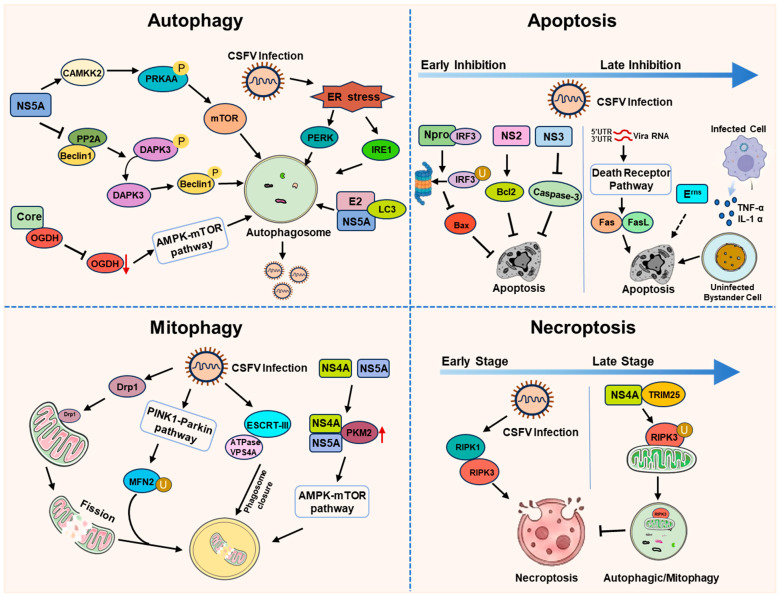
Strategies employed by CSFV to regulate host cell death through modulation of apoptosis, autophagy, mitophagy, and necroptosis.

CSFV employs a sophisticated strategy to manipulate host cell death pathways, striking a balance between promoting cell survival to facilitate viral replication and triggering death to aid dissemination. The interplay between autophagy, apoptosis, mitophagy, and necroptosis is critical for viral persistence and pathogenesis (Figure 4).

### 7.1. Autophagy

Autophagy is a conserved catabolic process essential for cellular homeostasis and innate immunity, degrading cytoplasmic components to recycle resources and eliminate intracellular pathogens [100,101]. However, numerous viruses, including CSFV, have evolved strategies to subvert this pathway to facilitate their own replication and persistence.

The infection of CSFV actively hijacks the autophagic machinery. It triggers endoplasmic reticulum (ER) stress, which subsequently activates the PERK and IRE1 pathways to induce autophagy [102]. The non-structural protein NS5A is a key inducer that functions by modulating specific axes, including the CAMKK2-PRKAA-MTOR axis [103] and the PP2A-DAPK3-Beclin 1 axis, where NS5A mediates the dissociation of PP2A from Beclin 1, leading to PP2A-mediated dephosphorylation and activation of DAPK3, which in turn phosphorylates Beclin 1 to trigger autophagosome formation [104]. Although the initiating signals vary, the outcome consistently leads to enhanced viral replication and establishment of infection.

Beyond NS5A, other viral proteins are instrumental in modulating autophagy. The core (C) protein interacts with and degrades α-ketoglutarate dehydrogenase (OGDH), regulating the AMPK-mTOR pathway to suppress the OGDH-dependent antiviral signaling and promote viral replication [93]. The envelope glycoprotein E2, along with NS5A, has been found to colocalize with the autophagosome marker LC3, suggesting that autophagosome-like structures may serve as sites for viral replication [105].

By co-opting these multiple autophagy-inducing pathways, CSFV not only secures metabolites and membrane structures for its replication complex but also manipulates the process to delay host cell apoptosis and dampen antiviral immune responses, ultimately leading to enhanced viral replication and the establishment of infection.

### 7.2. Apoptosis

CSFV’s manipulation of apoptosis is complex and stage-dependent, and exhibits significant differences between pathogenic and non-pathogenic strains. Pathogenic strains are more potent inducers of widespread lymphocyte apoptosis, a key driver of the immunosuppression and leukopenia characteristic of the acute disease, while attenuated strains may trigger less pronounced or delayed apoptotic responses [106].

To establish a productive infection, CSFV employs multiple strategies to inhibit early apoptosis in infected cells. The viral Npro protein not only acts as an antagonist of double-stranded RNA (dsRNA)-mediated IFN-α/β induction, but also potently suppresses dsRNA-triggered apoptosis [107]. The mechanism involves Npro’s interaction with interferon regulatory factor 3 (IRF3), targeting it for proteasomal degradation. By degrading IRF3, Npro effectively disrupts this IRF3/Bax-mediated apoptotic cascade, thereby preventing infected cell death [108]. Similarly, the NS3 protein interferes with the intrinsic apoptotic pathway by inhibiting the proteolytic activation of caspase-3 and modulating the activity of interferon regulatory factor 3 (IRF3), thereby suppressing a pro-apoptotic signal [103]. Furthermore, the NS2 protein has been shown to promote cell survival by upregulating anti-apoptotic proteins like Bcl-2 and resisting drug-induced apoptosis [109].

Conversely, CSFV, particularly pathogenic strains, actively induces apoptosis, especially in immune cells and during later stages of infection, to facilitate dissemination and evade immune clearance. The viral glycoprotein E^rns^ is a key inducer of lymphocyte apoptosis, contributing to the immunosuppression and leukopenia characteristic of the disease [110]. Additionally, the 5′ and 3′ untranslated regions (UTRs) of the CSFV genome can trigger apoptosis independently of viral protein coding sequences [111]. This direct induction is compounded by the activation of key death receptor pathways. Infection upregulates the expression of Fas and FasL, engaging the extrinsic apoptotic pathway in lymphocytes.

In vivo, apoptosis is observed both in infected cells and, significantly, in uninfected “bystander” cells. This bystander effect is mediated by soluble pro-apoptotic and pro-inflammatory factors. CSFV-infected macrophages secrete tumor necrosis factor-alpha (TNF-α) and interleukin-1 alpha (IL-1α), which can bind to death receptors on neighboring lymphocytes and other immune cells, propagating the apoptotic signal and leading to widespread lymphocyte depletion and cytokine storm [112,113,114].

### 7.3. Mitophagy

Mitophagy, a selective form of autophagy for degrading damaged mitochondria, is a central process manipulated by CSFV to inhibit the intrinsic apoptotic pathway. The virus first induces mitochondrial fission by promoting the translocation of Drp1 to mitochondria. Subsequently, CSFV activates the PINK1-Parkin pathway, leading to the ubiquitination and degradation of mitochondrial fusion mediators like Mitofusin 2 (MFN2). This tags the damaged mitochondria for encapsulation by autophagosomes, a process visualized by the colocalization of LC3 with mitochondria and lysosomes [115]. The non-structural protein NS4A, in conjunction with NS5A, upregulates pyruvate kinase M2 (PKM2) expression, activating the AMPK-mTOR pathway to induce mitophagy [94]. A groundbreaking study further demonstrated that CSFV hijacks the host ESCRT-III complex and ATPase VPS4A to promote the closure of phagophores (isolation membranes) during mitophagy, thereby accelerating the entire process [116]. By inducing mitophagy, CSFV achieves two critical objectives: clearing mitochondria that are prone to triggering apoptosis, and potentially replenishing the cell with metabolites to support viral replication.

### 7.4. Necroptosis

Necroptosis is a programmed form of inflammatory cell death, distinct from apoptosis, that is primarily mediated by the kinase activities of receptor-interacting protein kinases 1 and 3 (RIPK1 and RIPK3) [117]. This pathway can be activated as part of the host’s innate defense, and its dysregulation is often targeted by viruses. CSFV dynamically regulates this pathway in a temporal manner. In the early stage of infection, CSFV activates necroptosis in peripheral blood mononuclear cells (PBMCs) and the spleen, mediated by signaling complexes containing RIPK1 and RIPK3. This may facilitate initial viral spread or modulate the immune response. However, as the infection progresses to the late stage, the virus actively suppresses necroptosis to prevent excessive cell death and enable persistence. The NS4A protein plays a crucial role in this switch by interacting with the E3 ubiquitin ligase TRIM25, which targets RIPK3 for autophagic or mitophagy degradation, thereby markedly inhibiting necroptotic signaling [118].

## 8. Conclusions

The journey of CSFV, from initial attachment to final egress, demonstrates a sophisticated and multi-layered strategy to hijack and reprogram host cell machinery. This review has synthesized current knowledge, revealing several core themes in its pathogenesis. The interaction between CSFV and the host cell is initiated through a complex, multi-receptor entry process, involving molecules like HS, LamR, and CD46, which ensures robust cellular tropism. Upon entry, CSFV mounts a concerted attack on the host’s innate immune system. Viral proteins, particularly Npro, NS4A, NS5A, and NS5B, effectively dismantle critical signaling pathways such as the IRF3, NF-κB and interferon pathways, creating an immunologically stealth environment. Concurrently, the virus acts as a master metabolic engineer, strategically reprogramming the host’s lipid and glucose metabolism to fuel the massive biosynthetic demands of viral replication. Furthermore, CSFV exhibits exquisite control over cell fate, delicately balancing the induction of survival pathways like autophagy and mitophagy with the targeted triggering of death in immune cells, thereby facilitating both viral persistence and spread. The recent discovery of exosome-mediated viral transmission reveals a clever mechanism for evading humoral immunity, ensuring the virus can spread to neighboring cells undetected by neutralizing antibodies. Collectively, this integrated manipulation of cellular systems underpins the virus’s efficiency and its ability to establish persistent infections.

The intricate virus–host interface detailed herein reveals several promising vulnerabilities for therapeutic intervention. Key host dependency factors and virus–host protein interactions emerge as attractive targets. These include: (i) the pyrimidine synthesis enzyme DHODH, which is recruited by NS4A and is essential for viral replication; (ii) the exosome biogenesis and release machinery (e.g., Rab27a, MYO1B), crucial for antibody-evading viral spread; (iii) specific, well-characterized viral protein–host factor interactions that are central to immune evasion, such as the Npro-IRF3 and NS5A-NEMO axes, which could be disrupted by small molecules or peptides; and (iv) critical host metabolic enzymes like FASN, SCD1, and PKM2 that are commandeered to support the viral life cycle. Targeting these host factors or the interfaces of these essential interactions may provide a higher barrier to viral resistance compared to direct viral protein inhibitors.

Future research should bridge existing knowledge gaps and translate mechanistic insights into practical applications. Key priorities include employing advanced structural biology and interactomics to delineate critical virus–host interfaces, utilizing single-cell technologies and refined models to dissect the spatiotemporal dynamics of infection in vivo, and evaluating host-directed therapeutics (e.g., DHODH or metabolic inhibitors) alone or in combination with established strategies. Ultimately, building a predictive, systems-level understanding of the host–pathogen network will be essential for developing the next generation of broad-spectrum and durable countermeasures against CSFV.

## Figures and Tables

**Table 1:** Summary of the viral proteins of classical swine fever virus (CSFV) and their principal functions.

Protein	Size (kDa)	Key Features	Main Functions/Mechanisms	References
C	14	Nucleocapsid formation, virion assembly, enhances viral RNA synthesis, antagonizes innate immunity.	Binds viral RNA to form nucleocapsid. Interacts with NS5B polymerase to enhance replication. Binds host hemoglobin subunit β (HB) to inhibit the RIG-I interferon signaling pathway.	[9,10,11,12,13,14,15,16,17]
E^rns^	45	Receptor binding, ribonuclease (RNase) activity, induction of neutralizing antibodies.	Exists in membrane-anchored (for attachment) and soluble (secreted RNase) forms. Binds heparan sulfate and laminin receptor for cell adsorption.	[18,19,20,21,22,23,24,25,26]
E1	33	Mediates viral endocytosis.	Forms a disulfide-linked heterodimer with E2. As an embedded envelope protein, it is poorly immunogenic for neutralizing antibodies.	[27,28]
E2	55	Primary determinant of cell tropism, major inducer of neutralizing antibodies.	Binds multiple cellular receptors (e.g., CD46, MERKT). Forms heterodimers with E1 or homodimers. The primary target for diagnostic assays and subunit vaccine development.	[29,30,31,32]
Npro	23	Autocatalytic cysteine protease, antagonizes innate immunity.	Cleaves itself from the polyprotein. Targets IRF3 for proteasomal degradation, blocking I-IFN production.	[33,34,35,36]
p7	6–7	Ion channel activity.	Forms ion-conductive pores in membranes. Essential for efficient virus production and release.	[37,38,39,40,41,42]
NS2	53	Autoprotease.	Cleaves the junction between NS2 and NS3. The uncleaved NS2-NS3 precursor is essential for virion assembly.	[43,44,45,46,47]
NS3	80	Serine protease, RNA helicase and NTPase, essential for RNA synthesis and virion assembly.	Processes the viral polyprotein. Unwinds RNA duplexes and provides energy for replication. Core component of the membrane-associated replication complex.	[48,49,50,51]
NS4A	10	Cofactor for NS3 protease, RNA replication, modulation of cell metabolism/death.	Localizes to ER and mitochondria; interacts with DHODH to boost UMP production for viral RNA synthesis.	[49,52,53,54]
NS4B	35	Replication complex formation, inhibits TLR3 signaling pathway.	Localizes to Golgi apparatus. Suppresses TLR3-mediated induction of IFN-β and pro-inflammatory cytokines.	[49,56]
NS5A	58	RNA-binding regulatory protein.	Regulates viral RNA synthesis and IRES-mediated translation. Interacts with multiple host factors (e.g., PKM2, Rab14) to rewire metabolism and promote viral propagation.	[57,58]
NS5B	77	RNA-dependent RNA polymerase (RdRp), modifies host epitranscriptome.	Catalytic core of the viral replication complex. Stabilizes m6A methyltransferase METTL14 to promote degradation of TLR4 mRNA, suppressing antiviral signaling.	[59,60,61]

**Table 2:** Summary of host cellular receptors/attachment factors for CSFV entry.

Receptor/Attachment Factor	Interacting Viral Protein	Major Expressing Cells/Tissues	Function	Essential for In Vivo Infection?	References
HS	E^rns^	Ubiquitous on cell surfaces	Receptor	No	[19,20]
LamR	E^rns^	Ubiquitous	Receptor	/	[21]
CD46	/	Ubiquitous	Receptor	/	[62]
Integrin β3	/	Platelets, endothelial cells, macrophages, etc.	Attachment factor	/	[63]
Annexin II	E2	Ubiquitous	Attachment factor	/	[64]
MERTK	E2	Immune cells, epithelial cells, etc.	Attachment factor	/	[65]
ADAM17	E2	Immune cells, endothelial cells and tumor cells	Critical attachment factor	Essential in primary porcine fibroblast	[66]
Vinculin	/	Focal adhesions, cell junctions	Possible attachment factor	/	[21]
LDLR	/	Ubiquitous	Putative indirect factor	No	[67]

“/” in the table means that this has not been demonstrated.

## Data Availability

No new data were created or analyzed in this study. Data sharing is not applicable to this article.
